# Effectiveness and Safety of Acupuncture for Nausea and Vomiting in Cancer Patients: A Systematic Review and Meta-Analysis

**DOI:** 10.3390/medicina61071287

**Published:** 2025-07-17

**Authors:** Sung-A Kim, Sujung Yeo, Sabina Lim

**Affiliations:** 1 Department of Clinical Korean Medicine, Graduate School, Kyung Hee University, Seoul 02447, Republic of Korea; 777tjddk@khu.ac.kr; 2 Epidemiology Group, Aberdeen Centre for Arthritis and Musculoskeletal Health, University of Aberdeen, Aberdeen AB24 3FX, UK; 3 Research Institute of Korean Medicine, Sangji University, Wonju 26339, Republic of Korea; 4 Research Group of Pain and Neuroscience, WHO Collaborating Center for Traditional Medicine, East-West Medical Research Institute, Kyung Hee University, Seoul 02447, Republic of Korea; 5 Department of Meridian and Acupoint, College of Korean Medicine, Kyung Hee University, Seoul 02447, Republic of Korea

**Keywords:** cancer, nausea, vomiting, acupuncture, systematic review, meta-analysis

## Abstract

*Background and Objectives*: Nausea and vomiting (NV) are common and distressing adverse effects among cancer patients undergoing treatment. Despite the widespread use of pharmacological antiemetics, these medications are often insufficient for controlling nausea and may cause medication interactions and side effects. Acupuncture has been proposed as a complementary therapy; however, the comprehensive analysis of its effects on NV across all emetogenic cancer treatments remains limited. This systematic review and meta-analysis aimed to evaluate the effectiveness and safety of acupuncture in managing NV in cancer patients undergoing chemotherapy, radiotherapy, or surgery. *Materials and Methods*: We conducted a comprehensive search across three electronic databases and two clinical registry platforms from inception to December 2024. Randomized controlled trials (RCTs) evaluating acupuncture for NV in cancer patients were included. Risk ratios (RRs) and 95% confidence intervals (CIs) were calculated using a random-effects model. Safety outcomes were assessed based on the Common Terminology Criteria for Adverse Events (CTCAE). *Results*: Seventeen RCTs met the inclusion criteria, with twelve studies included in the meta-analysis. Acupuncture did not demonstrate significant effects on acute nausea (RR: 0.98; 95% CI: 0.84–1.15; *p* = 0.80) or acute vomiting (RR: 0.93; 95% CI: 0.65–1.32; *p* = 0.67). However, it significantly reduced delayed vomiting (RR: 0.76; 95% CI: 0.61–0.95; *p* = 0.02). Subgroup analysis demonstrated significant effects when acupuncture was administered for at least five days (RR: 0.56; 95% CI: 0.39–0.81; *p* = 0.002). The most frequently used acupoints were PC6, ST36, CV12, LI4, LR3, and ST25. No serious adverse events related to acupuncture treatments were reported, with only minor AEs such as localized bleeding and mild bruising observed. *Conclusions*: Acupuncture represents a safe and effective complementary therapy for managing delayed vomiting in cancer patients receiving emetogenic treatments. Clinicians can anticipate optimal benefits from at least five days of treatment, particularly using acupoints PC6, ST36, CV12, LI4, LR3, and ST25. Further high-quality studies are needed to establish standardized treatment regimens and explore its comprehensive effects on NV.

## 1. Introduction

Nausea and vomiting (NV) are prevalent and distressing symptoms experienced by over 80% of cancer patients undergoing chemotherapy, radiotherapy, or surgery [1]. These symptoms not only compromise patients’ quality of life but can also lead to serious complications including dehydration, malnutrition, and decreased treatment adherence [2]. Current pharmacological interventions, including 5-HT3 and NK-1 receptor antagonists, have demonstrated effectiveness in controlling vomiting; however, they are less effective in alleviating nausea [3]. Additionally, these medications may cause side effects including sedation, constipation, and, in rare cases, cardiac arrhythmias [4]. Consequently, many patients seek effective, safe, and sustainable multidisciplinary approaches to alleviate symptoms and enhance overall well-being.

Acupuncture has emerged as a promising non-pharmacological therapy for managing NV among cancer patients. Acupuncture stimulates various physiological responses, including the release of endorphins and the modulation of the autonomic nervous system, which can alleviate NV symptoms [5]. Notably, the PC6 (Neiguan) acupuncture point has been shown to modulate the vomiting center in the brainstem, providing relief from NV [6]. Beyond its biological effects, cancer patients who have received regular acupuncture treatments have reported significant improvements in physical complaints, psychological well-being, and enhanced coping strategies [7]. Furthermore, acupuncture has been recognized as being as safe as non-invasive sham acupuncture or active controls in oncological patients [8].

However, while previous meta-analyses have investigated the effectiveness of acupuncture, most studies have predominantly focused on chemotherapy-induced nausea and vomiting (CINV), often overlooking NV resulting from other cancer therapies such as radiotherapy and surgery [9]. Moreover, existing reviews have not adequately addressed the safety profile of acupuncture and exhibit heterogeneity in results due to variability in study designs, acupuncture techniques, and outcome measures. As acupuncture continues to be integrated into cancer care, it is essential to establish robust evidence supporting both its effectiveness and safety, especially among diverse and complex cancer populations [10].

The primary objective of this systematic review and meta-analysis was to comprehensively evaluate the effectiveness and safety of acupuncture in managing NV in cancer patients undergoing various emetogenic treatments including chemotherapy, radiotherapy, and surgery. In addition, we thoroughly analyzed acupuncture protocols according to the STandards for Reporting Interventions in Clinical Trials of Acupuncture (STRICTA) guidelines to provide clinicians with evidence-based guidance on acupuncture utilization, ultimately improving patient outcomes in cancer care.

## 2. Materials and Methods

This systematic review and meta-analysis were performed in compliance with the Preferred Reporting Items for Systematic Reviews and Meta-Analyses (PRISMA) guidelines. We registered the protocol in the PROSPERO database (registration number: CRD42022333811) and published the study protocol [11].

### 2.1. Criteria for Inclusion and Exclusion

#### 2.1.1. Types of Studies

Only RCTs evaluating the effectiveness and safety of acupuncture treatments for nausea and vomiting (NV) in cancer patients were included. We systematically included cancer patients undergoing oncology-related treatments (such as chemotherapy, radiotherapy, or surgery) known to trigger NV.

#### 2.1.2. Types of Participants

Studies encompassing patients of all types of cancer (e.g., solid tumors, hematological malignancies), regardless of age, gender, nationality, ethnicity, or underlying cause of their symptoms, were eligible.

#### 2.1.3. Types of Interventions

We included studies with needle-based acupuncture interventions such as manual acupuncture, electroacupuncture, intradermal acupuncture, auricular acupuncture, and laser acupuncture. All these involved skin puncture through an acupuncture needle or laser. This included various methods such as manual acupuncture (direct insertion of needles), electroacupuncture (needles with electronic stimulation), intradermal acupuncture (short needles embedded with tape for several days), auricular acupuncture (needles placed in the ear), and laser acupuncture (non-needle-based but still involving point stimulation). We included acupuncture sessions conducted either prior to or following cancer treatment. Non-needle-based acupuncture treatments, such as acupressure, cupping, moxibustion, transcutaneous electrical acupoint stimulation, and other similar methods, were excluded. These therapies do not involve skin puncture or stimulation with needles.

#### 2.1.4. Types of Comparators

Comparison interventions included active therapies, sham acupuncture (non-penetrating sham acupuncture, pseudoacupuncture interventions, sham acupuncture at non-acupoints, and needling at inappropriate acupoints), and no treatment. Studies where acupuncture was used either alone or in combination with therapies that were the same as the comparison intervention were included.

#### 2.1.5. Types of Outcome Measures

Studies were included if they reported both effectiveness and safety outcomes. The primary outcome for effectiveness was the incidence of NV, which was determined by analyzing completely controlled events at different endpoints. Secondary outcomes with various patient-reported scales included the Multinational Association of Supportive Care in Cancer Antiemesis Tool (MAT), Functional Living Index Emesis tool (FLIE), the Rhodes Index of Nausea, Vomiting, and Retching (RINVR), numeric rating scale (NRS), and other validated instruments for assessing both symptomatic and preventive effects of acupuncture. Safety outcomes included the types, grade, and incidence of adverse events.

### 2.2. Literature Searches

Studies published up to December 2024 were retrieved from the following three databases: MEDLINE via PubMed, EMBASE via Ovid, and the Cochrane Central Register of Controlled Trials. Two clinical registries, the National Institutes of Health Clinical Registry (ClinicalTrials.gov) and the World Health Organization International Clinical Trials Registry Platform, were also screened to mitigate the risk of publication bias. To identify any potentially missing eligible studies, we manually scanned the reference lists of other systematic reviews using the following search terms: cancer, tumor/tumor, neoplasm, nausea, vomiting, emesis, acupuncture, and RCTs. These terms were utilized alone or in combinations using “and,” “or”. The search strategy was tailored for each respective database. There was no language restriction.

### 2.3. Data Selection

**Figure 1:**
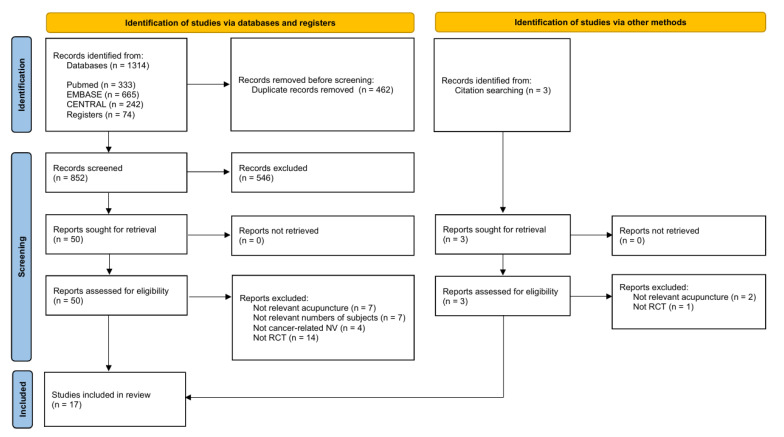
Study flow chart.

The titles and abstracts of the studies were reviewed by two independent researchers (S.A.K. and S.B.N.L.) following the retrieval of all eligible studies in EndNote 21 (Clarivate Analytics), with duplicate publications being removed. The full texts of all potentially eligible studies were retrieved. Any disagreements regarding data selection were addressed by an arbiter (Y.S.J.). A PRISMA flow diagram shows the study search and screening process (Figure 1).

### 2.4. Data Extraction

Data extraction was conducted independently by two reviewers (S.A.K. and S.B.N.L.) using a pre-specified and standardized data extraction form. This form was pilot-tested on a subset of studies to ensure consistency in capturing relevant information, such as on participant characteristics, interventions, control conditions, outcome measures, and adverse events. In instances of incomplete or unclear data, we reached out to the original study authors for clarification. The extracted data included the total number of patients randomized, specifics of the acupuncture intervention, and the primary and secondary outcomes reported by each study. In light of the complexity of acupuncture intervention, we conducted an additional data extraction of the treatment protocol in accordance with the STRICTA guideline [12]. Disagreements between the reviewers were addressed through discussion. If consensus could not be achieved, a third reviewer (Y.S.J.) was consulted.

### 2.5. Quality Assessment of Included Studies

Two authors (S.A.K. and S.B.N.L.) independently evaluated the risk of bias (ROB) using the Cochrane Collaboration assessment method [13]. The biases assessed included selection, performance, attrition, detection, reporting, and other potential sources of bias. Judgments regarding these biases were categorized as low, high, or unclear. The results were summarized graphically through the plotting of bias assessments. Any discrepancies in bias assessment were resolved by the arbiter (Y.S.J.).

### 2.6. Data Analysis

A meta-analysis of aggregated data was conducted to analyze the incidence of NV and adverse events as individual patient data was not accessible. A meta-analysis was conducted using data from dichotomous events of nausea or vomiting occurring in acute (within 24 h) and delayed phases (24 h or later), compared to sham acupuncture or usual care. In multi-arm studies, when multiple control or intervention groups were present, double counting was avoided by appropriately selecting the most relevant comparisons. If more than two groups were relevant, groups were combined to create a single pair-wise comparison [14]. For the analysis of complete control effect of the treatment, we included any instance of nausea or vomiting as an event. A random-effects model was employed to calculate risk ratios (RRs) with 95% confidence intervals (CIs), due to the substantial statistical heterogeneity observed among the included studies. An RR less than 1 indicated that the acupuncture treatment group experienced more effective complete control of NV. Heterogeneity was evaluated for quantifying inconsistency using the I^2^ test and statistical heterogeneity was identified through the standard χ^2^ test. If the I^2^ value > 50% or the *p*-value was <0.100, studies were considered to have significant heterogeneity, which prompted the conduct of subgroup analyses to investigate potential causes. Statistical significance was defined as *p* < 0.05. If the data from the trials were not appropriate to be merged for meta-analysis, we conducted a descriptive analysis instead. We did not conduct a sensitivity analysis due to the limited number of available studies included in meta-analysis. Safety analysis was conducted to classify the grade of AEs according to the Common Terminology Criteria for Adverse Events (CTCAE): Grade 1 (mild or asymptomatic, only clinical observations only), Grade 2 (moderate, local or noninvasive interventions required), Grade 3 (severe, medically significant by not immediately life-threatening), Grade 4 (life-threatening, urgent intervention required), and Grade 5 (death related to AE) [15]. To assess reporting biases, funnel plots were analyzed by Egger’s test when more than ten studies were included for nausea and vomiting. Data analyses were performed using R (R Version 4.3.1, Windows) and RevMan Version 5.4 software (Cochrane Collaboration, Oxford, UK).

## 3. Results

### 3.1. Study Characteristics

A total of 1314 publications were identified from databases and clinical registries, with three additional records identified from other sources. After removing duplicate records, we screened the titles and abstracts of 852 studies. Fifty full-text articles were subsequently assessed for eligibility, resulting in the inclusion of fifteen studies in this review. The search procedures are presented in the PRISMA 2020 flowchart in Figure 1.

These seventeen studies included a total of 2036 patients, with sample sizes ranging from 32 to 324. The types of cancer varied; seven studies focused exclusively on patients with a single cancer type (e.g., breast, colorectal, gynecologic, or lung cancer) whereas ten studies comprehensively included multiple carcinoma types. The causes of NV included chemotherapy, blood stem cell transplantation, surgery, and radiotherapy. Among these causes, 14 studies (82.4%) investigated patients experiencing CINV, with eight of these studies reporting on the type of anticancer drugs utilized.

Among the seventeen studies, four studies employed a three-arm RCT design, two studies applied a four-arm design, and eleven studies adopted a two-arm RCT design. Within the six studies featuring three-arm or four-arm RCT design, two studies investigated acupuncture therapy as an adjunct to pharmacotherapy compared to both sham acupuncture combined with pharmacotherapy and pharmacotherapy alone. The remaining four studies compared multiple intervention groups based on different treatment sessions or timelines, such as before versus after chemotherapies. Regarding intervention methods, seven studies (41.2%) used manual acupuncture, another eight studies (47.1%) utilized electroacupuncture, and two studies (11.8%) implemented intradermal acupuncture. Additionally, complementary pharmacotherapies were administered alongside acupuncture treatment in 15 studies (88.2%). For the control groups, seven studies (41.2%) used sham acupuncture, five studies (29.4%) incorporated pharmacotherapy, two studies (11.8%) employed only usual care, one study (5.9%) used a no-treatment control, and two studies (11.8%) utilized a three-arm design, featuring two control groups that included both sham acupuncture and pharmacotherapy. Among nine studies using sham acupuncture, the control groups varied from non-penetrating sham acupuncture to minimal acupuncture at non-acupoint sites.

Outcome measures included the incidence of NV, with additional patient self-assessment scales like the effective rate; numeric rating scale (NRS); Rhodes Index of Nausea, Vomiting, and Retching (RINVR); and Multinational Association of Supportive Care in Cancer Antiemesis Tool (MAT). Excluding two studies that did not report adverse events (AEs), it was found that seven studies reported no AEs, eight studies reported only minor-to-moderate AEs, and no serious AEs were observed. Most studies demonstrated the symptomatic effectiveness of acupuncture, particularly in managing delayed vomiting. While a few studies explored the preventive effects of acupuncture on NV, the overall evidence regarding prevention remained inconclusive. The main findings were identified as positive in thirteen studies while four studies concluded with unclear or negative results (Table 1).

### 3.2. Acupuncture Treatment Protocol Analysis

**Figure 2:**
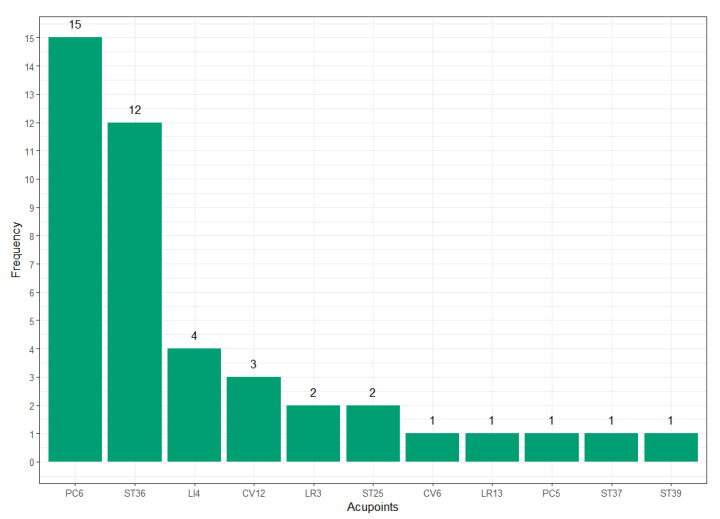
Acupuncture point frequency.

A total of 11 acupuncture points were used across the 17 trials. Each point was used 1–15 times in various combinations. The most frequently used point was PC6, which was prescribed in 15 of the 17 studies. The second most frequently used point was ST36, which was selected in 12 studies. The following points were infrequently used in two or three studies: CV12, LI4, LR3, and ST25. Points rarely used in one study were CV6, LR13, PC5, ST37, and ST39 (Figure 2). The sites of acupuncture points with the anatomical location terms are presented in Appendix A Table A1 [33].

Needle stimulation types included manual acupuncture (41.2%), electroacupuncture (47.1%), and intradermal acupuncture treatment (11.8%). In electroacupuncture treatment, the frequency used in most studies was 2–20 Hz, and one study used a mixed frequency of 2/100 Hz. De-qi was elicited in fourteen studies using techniques such as rotation, twirling, lifting, or thrusting whereas three studies did not report whether it was induced. The number of needle insertions varied between two and fourteen. For the needle retention time, 30 min was most frequently used (47.1%), and the next most used was 20 min (29.4%). Exceptionally, intradermal acupuncture was maintained for 3 or 4 days. For patients with CINV, the treatment time ranged from 10 min to 2 h, most commonly before chemotherapy, with 30 min being the most frequently used duration. In other patients, acupuncture treatment was performed 1 and 2 days after surgery, whereas it was implemented before or after the radiotherapy sessions. The number of treatment sessions varied from one to twelve (Table 2).

### 3.3. Quality of Included Studies

**Figure 3:**
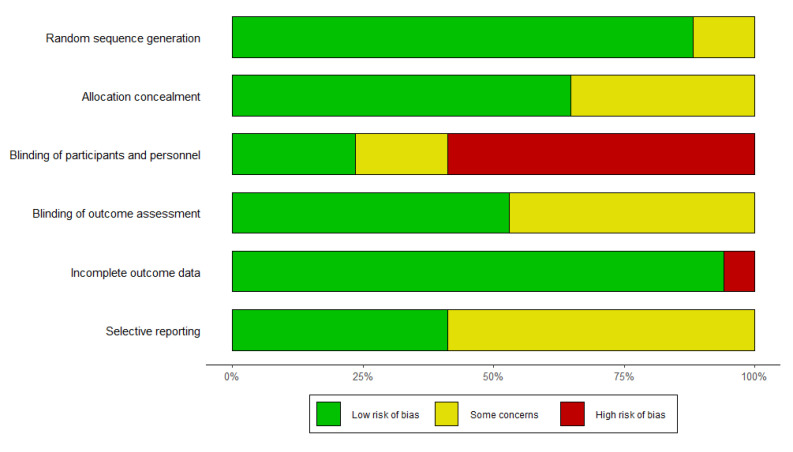
Bias assessment of acupuncture treatment.

The overall results of the ROB analysis are summarized in Figure 3. In terms of random sequence generation, fifteen studies had a low risk while two studies had an unclear risk. For allocation concealment, eleven studies had a low risk and six studies had an unclear risk. Regarding the blinding of patients and personnel, four studies had a low risk, three studies had an unclear risk, and ten studies had a high risk. Concerning the blinding of the outcome assessment, nine studies had a low risk and eight studies had an unclear risk. With respect to incomplete outcome data, sixteen studies were at low risk and one study had a high risk. In the area of selective reporting, seven studies were at low risk and ten studies were at unclear risk. All studies had a low ROB with regard to other biases (e.g., conflict of interest, deviations from the protocol, or problems related to cross-over design).

Funnel plots were not analyzed because fewer than 10 studies each were available for nausea and vomiting.

### 3.4. Effects of Acupuncture on Nausea

**Figure 4:**
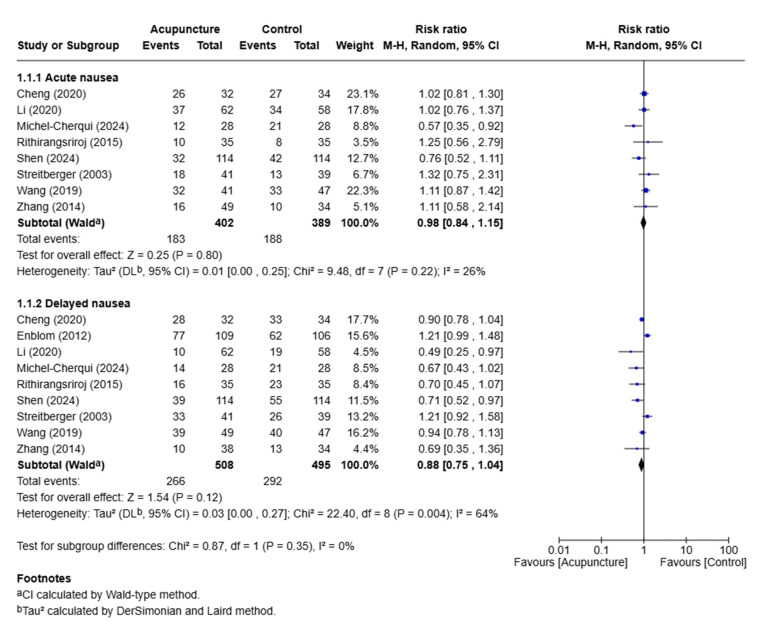
Meta-analysis of acupuncture treatment for patients with nausea [17,20,22,24,26,27,28,31,32].

A meta-analysis was conducted to evaluate the effectiveness of acupuncture treatment on acute (within 24 h) and delayed phases (24 h or later) of nausea in comparison to sham acupuncture or usual care. In the acute phase, acupuncture had no significant effect on patients with nausea (RR: 0.98; 95% CI: 0.84 to 1.15; *p* = 0.80; I^2^ = 26%; 8 RCTs; *n* = 791). In the delayed phase, acupuncture had no significant effect on patients with nausea (RR: 0.88; 95% CI: 0.75 to 1.04; *p* = 0.12; I^2^ = 64%; 9 RCTs; *n* = 1003) (Figure 4).

### 3.5. Effects of Acupuncture on Vomiting

**Figure 5:**
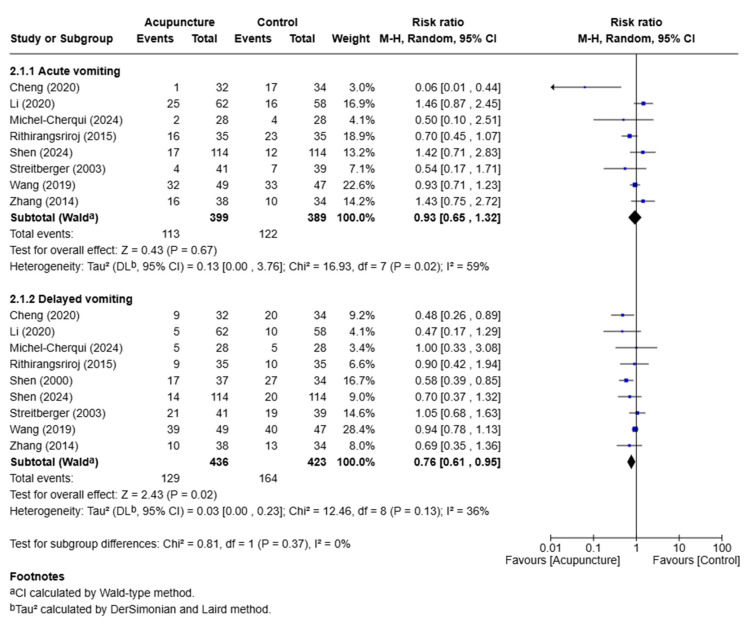
Meta-analysis of acupuncture treatment for patients with vomiting [16,17,22,24,26,27,28,31,32].

A meta-analysis was conducted to evaluate the effectiveness of acupuncture treatment during both the acute and delayed phases of vomiting compared to sham acupuncture or usual care. In the acute phase, acupuncture had no significant effect on patients with vomiting (RR: 0.93; 95% CI: 0.65 to 1.32; *p* = 0.67; I^2^ = 59%; 8 RCTs; *n* = 788). In the delayed phase, acupuncture had a significant effect on patients with vomiting (RR: 0.76; 95% CI: 0.61 to 0.95; *p* = 0.02; I^2^ = 36%; 9 RCTs; *n* = 859) (Figure 5).

**Figure 6:**
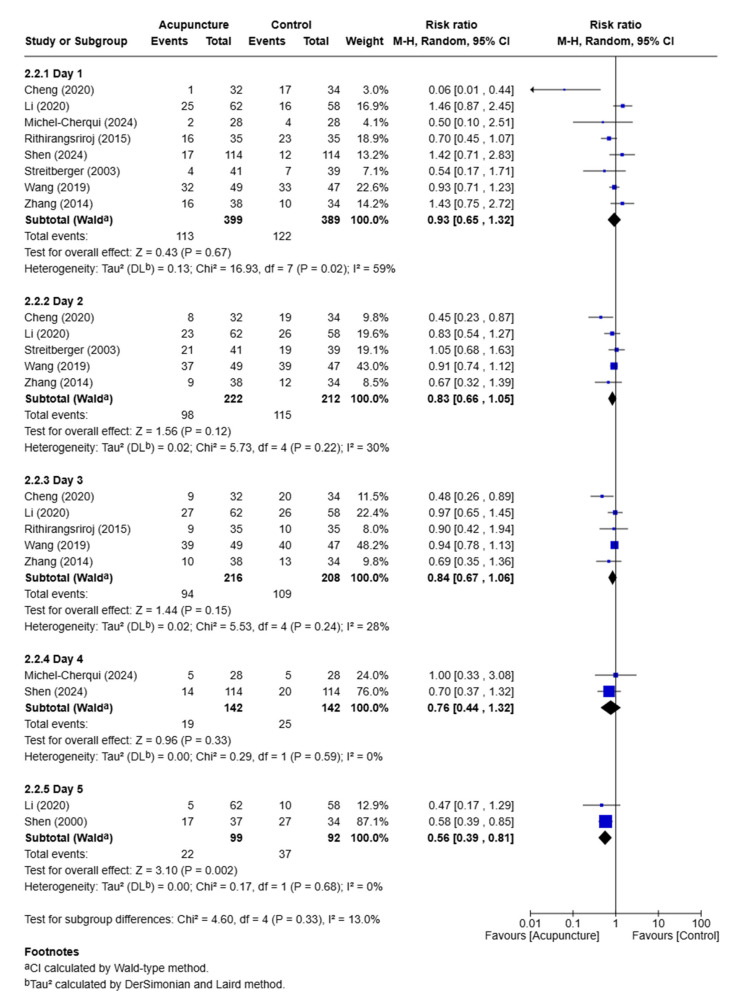
Daily meta-analysis of acupuncture treatment for patients with vomiting [16,17,22,24,26,27,28,31,32].

A day-by-day subgroup meta-analysis of acupuncture treatment was performed for the subgroup analysis. Studies were categorized into groups for days 1, 2, 3, 4, and 5 or more. On day 1, defined as the acute phase, the results aligned with those of the acute phase of vomiting. On days 2, 3, and 4, acupuncture had no significant effect on patients with vomiting (RR: 0.83; 95% CI: 0.66 to 1.05; *p* = 0.12; I^2^ = 30%; 5 RCTs; *n* = 434, for day 2; RR: 0.84; 95% CI: 0.67 to 1.06; *p* = 0.15; I^2^ = 28%; 5 RCTs; *n* = 424, for day 3; RR: 0.76; 95% CI: 0.44 to 1.32; *p* = 0.33; I^2^ = 0%; 2 RCTs; *n* = 284, for day 4). On day 5 or beyond, two studies indicated that acupuncture had a significant effect on patients with vomiting (RR: 0.56; 95% CI: 0.39 to 0.81; *p* = 0.002; I^2^ = 0%; 2 RCTs; *n* = 92) (Figure 6).

### 3.6. Safety of Acupuncture

Regarding the safety of acupuncture, six studies did not report adverse events (AEs), three studies reported no AEs, and eight studies reported AEs. Among the eleven studies that documented AEs, eight studies reported AEs from both intervention and control groups. Due to the limited information available, we were unable to analyze the incidence rate; however, we narratively summarized the types, grades, and incidence of AEs in Appendix A Table A2.

According to the CTCAE, a total of 192 AEs in intervention groups (eleven RCTs, *n* = 620) and 116 AEs in control groups (eight RCTs, *n* = 516) were classified as Grade 1, minor AEs. No additional interventions were required for these AEs. Localized bleeding around needle insertion points and needling pain were commonly reported, followed by constipation, tiredness, symptom aggravation, etc. One study reported Grade 2, moderate AEs, which involved symptom exacerbation in participants experiencing CINV, with 17 AEs in the intervention group and 22 AEs in the control group requiring rescue medication. One study reported five cases of serious AEs due to cancer; however, the author did not specify what they were, merely noting that they were not associated with acupuncture treatment (Appendix A Table A2).

## 4. Discussion

Seventeen RCTs were included for qualitative synthesis and ten RCTs were analyzed for quantitative synthesis. The overall effect of acupuncture on NV was not significant in the acute phase. However, there was a significant reduction in delayed vomiting when compared to sham acupuncture or no treatment. No serious or infectious AEs related to acupuncture treatments were reported, supporting the safety of acupuncture. These findings highlight that acupuncture may serve as an effective and safe complementary therapy for managing delayed symptoms in cancer patients.

This study presented several strengths. Firstly, this study applied rigorous outcome measures in a meta-analysis, prioritizing complete symptom control over partial improvement, and identified the long-term effects of acupuncture. While this conservative approach may have contributed to smaller overall effect sizes and non-significant pooled results, it is noteworthy that some individual studies reported significant improvements in secondary outcomes using validated scales such as the Visual Analogue Scale (VAS); the Rhodes Index of Nausea, Vomiting and Retching (RINVR); and the MASCC Antiemesis Tool (MAT), even when complete symptom control was not achieved [18,29,32]. This suggests that acupuncture may provide meaningful clinical benefits in reducing symptom severity, improving the overall quality of life, and alleviating subjective distress that are not captured by binary incidence measures alone. Instead, subsequent subgroup analyses stratified by treatment duration addressed the heterogeneity of the studies and confirmed the effectiveness. This aligned with previous research indicating that acupuncture may exert more pronounced therapeutic effects over time, as opposed to producing immediate symptomatic relief [34]. Although we cannot entirely rule out the possibility of spontaneous remission over time, the observed significant impact of acupuncture on delayed vomiting, rather than on acute symptoms, may be attributable to the distinct and complex pathophysiology underlying these two phases of NV. Acute symptoms are primarily mediated by the rapid release of serotonin (5-HT3) in the small intestine, which are effectively controlled by standard antiemetics, particularly 5-HT3 receptor antagonists, which immediately block these fast-acting neurotransmitter pathways [35]. Conversely, delayed vomiting involves the prolonged activation of substance P and neurokinin-1 receptor pathways and dysregulation of the hypothalamic–pituitary–adrenal axis [36]. These processes extend beyond simple neurotransmitter release, encompassing broader neuroendocrine and inflammatory cascades that develop over time. Acupuncture influences serotonin pathways differently from mechanisms involved in the acute phase, potentially by modulating serotonin synthesis and metabolism rather than receptor blockade [37]. Consequently, the preferential effectiveness of acupuncture in regulating gastric motility and restoring normal gastrointestinal function may be particularly relevant for managing delayed vomiting, in contrast to the more immediate, peripheral mechanisms that primarily influence acute NV [38]. This has critical implications for clinicians in determining treatment methodologies, particularly for patients experiencing breakthrough delayed symptoms despite optimal pharmacological management.

Secondly, we systematically extracted data on treatment regimens from each of the included studies. Among the acupuncture points prescribed, PC6 was the most frequently used, followed by ST36. Additional points, including CV12, LI4, LR3, and ST25, were also used multiple times across the total eleven acupuncture points analyzed. In previous network analyses, the acupuncture points CV12, ST25, ST36, and LR3 were identified as the main acupuncture points for treating gastrointestinal dysfunction. This was achieved through the modulation of the 5-hydroxytryptamine system, reductions in visceral hypersensitivity, the enhancement of gastrointestinal motility, the improvement of mucosal permeability, and the promotion of neuropeptide hormone secretion [39,40]. Recent findings regarding ST36 have newly determined that modulation of the vagal–adrenal axis via somatosensory–autonomic reflexes may further alleviate NV [41]. These acupuncture points are also frequently applied to address fatigue, pain, sleep disorders, and psychological symptoms in cancer patients. Since these comorbidities are also recognized as risk factors for NV, concurrently managing these symptoms may improve NV control [39,42,43]. Furthermore, these acupuncture points have been confirmed to have a low incidence of AEs due to their anatomical locations in the abdomen or limbs [44]. Therefore, we affirmed both the effectiveness and safety of these points, suggesting that their combined application could be a valuable treatment option in clinical practice.

Third, we raised concerns regarding the adequacy of several acupuncture parameters in the protocols, specifically in terms of dosage. Establishing a treatment regimen with sufficient dosage tailored for specific symptoms is critical in RCTs. Moreover, as acupuncture comprises a complex and multifaceted intervention, numerous components of acupuncture needling procedures, such as the insertion depth, needle size, treatment period, frequency, and number of needles, must be considered. In this review, most studies implemented treatment across five sessions with fewer than ten needles. Even for conditions like pain, recommended dosages are known to be at least two sessions per week for a duration of six to twelve weeks [45]. Our findings suggest that the dosage of acupuncture treatment may not have been adequate to attain an optimal therapeutic response. The subtherapeutic dosing incorporated in the included RCTs may have been insufficient to elicit a measurable effect, particularly concerning acute NV symptoms. Additionally, the absence of standardization in acupuncture protocols, including variation in acupoint selection, the number of treatment sessions, and the number of needle insertions, may have diluted the treatment effects.

Fourth, we found an inadequate reporting of safety outcomes in these included studies. Only eight out of the seventeen included studies reported AEs, and even among those, two studies did not report AEs in the control groups. Moreover, AE descriptions were generally limited to symptom listings and were not reported using structured criteria such as the CTCAE, a widely accepted framework in oncology trials. Although no serious AEs were reported, the lack of comprehensive AE reporting is particularly concerning in cancer populations, who are often more vulnerable to complications. A standardized and thorough approach to safety monitoring is essential in future research in full accordance with CTCAE, including the type, severity, and causality of AEs across all study arms. This would not only enhance transparency but also facilitate the accurate assessment of acupuncture’s safety profile in this sensitive population.

This study had several limitations. First, we did not find any significant effects of acupuncture on nausea or vomiting in the acute phase. Our meta-analysis performed on the incidence of NV reflected only complete symptom control. Although there were a few validated outcome measurements, such as the RINVR and MAT, most of the included studies did not apply these outcome measures. Incorporating these outcomes, which evaluate symptom frequency, duration, and patient discomfort, would provide researchers with valuable data and demonstrate gradual improvements through numerical scoring. Additionally, a more comprehensive approach that considers other psychological or symptom-related factors (e.g., anxiety, sleep disorders, fatigue, or reducing AEs from pharmacotherapy) as secondary outcome measures would be valuable for understanding cancer patients’ experiences. We also attempted subgroup analyses according to concomitant treatments and timelines of treatment implementation. However, we could not find any significant results or reductions in heterogeneity from these analyses. Second, we focused not only on preventive and symptomatic effectiveness of acupuncture but also on its safety. While we initially aimed to analyze the incidence rate of AEs using a forest plot, the majority of the studies included did not adequately report the information related to AEs. Considering that cancer patients face risks associated with immunocompromised states, a fear of needles, and possibilities of neuropathy and edema, it would be necessary to review the safety of acupuncture in cancer populations minutely. Future clinical trials should evaluate the types and grade of AEs in detail, using standardized scales such as the CTCAE [15]. Third, substantial heterogeneity in acupuncture treatment protocols and control group designs may have contributed to an underestimation of treatment effects. In addition, most importantly, 14 out of the 17 included trials (82.4%) administered acupuncture in combination with standard antiemetic treatments, which limited the ability to assess the independent therapeutic effects of acupuncture. While a sensitivity analysis excluding combination therapy arms would have been informative for isolating the standalone efficacy of acupuncture, this was not feasible due to the small number of monotherapy trials available. Similarly, although meta-regression analysis could provide valuable insights into potential treatment effect modifiers such as the number of needle insertions, depth of insertion, or needle stimulation techniques, our sample size was insufficient to conduct a statistically robust analysis. Future systematic reviews should be designed with sufficient data to enable subgroup and sensitivity analyses comparing monotherapy versus combination approaches, as well as meta-regression to better understand the influence of specific acupuncture parameters on treatment outcomes.

Based on these identified research gaps, this review highlights several areas requiring attention in future research to strengthen the evidence base and clinical application of acupuncture for cancer-related NV. First, future studies should focus on various emetogenic factors. Although we attempted to comprehensively assess emetic factors affecting cancer patients, the majority of studies concentrated on CINV, despite the fact that cancer patients frequently experience NV from other causes, including radiotherapy, surgery, and tumor invasion, particularly in gastrointestinal or advanced-stage cancers. Furthermore, all studies primarily investigated acute or delayed phases of NV, leaving anticipatory NV largely unexamined. Future studies should explore acupuncture’s potential in preventing not only acute and delayed NV but also anticipatory NV, which remains a major and distressing challenge for cancer patients [46]. This limited scope represents a significant research gap, and future studies should expand their inclusion criteria to evaluate acupuncture’s potential across a broader range of NV etiologies to better reflect real-world clinical settings. In addition, overall methodological limitations and heterogeneity in study designs should be improved. A substantial proportion of studies demonstrated high or unclear risk of bias in the domains of participant/personnel blinding and outcome assessment. While blinding is inherently challenging in acupuncture research, methodological strategies such as the use of independent outcome assessors, centralized data collection, the assessment of blinding, or validated sham techniques can help mitigate performance and detection biases [47]. Regarding improvements in reporting methodology, the detailed documentation of trial processes and safety monitoring according to well-established guidelines, including the CONsolidated Standards Of Reporting Trials (CONSORT), STRICTA, and CTCAE, are essential for enhancing the credibility and reproducibility of future trials. Furthermore, the optimal acupuncture regimen for cancer-related NV remains still unclear despite numerous trials having been conducted. Key dosage parameters, including the number of sessions and needle retention times, varied widely and were often below thresholds suggested by previous research as therapeutically effective. This highlights the need for well-designed dose–response studies and consensus-driven guidelines to standardize acupuncture protocols, ensuring both efficacy and practical applicability. Finally, based on our comprehensive analysis across different cancer types and emetogenic factors, future clinical trials can incorporate long-term follow-up periods in specific oncological populations and treatment settings.

## 5. Conclusions

This systematic review and meta-analysis provided evidence that acupuncture treatment is an effective and safe complementary therapy for delayed vomiting in cancer patients undergoing emetogenic therapies. However, its effects on acute NV remain inconclusive, and we encountered challenges in determining the adequate acupuncture treatment dosages for optimal therapeutic responses. Clinicians should consider incorporating acupuncture into a multimodal approach, particularly for patients experiencing delayed emesis. Further high-quality research should focus on refining acupuncture parameters, including robust AE reporting, and investigating its preventive potential for anticipatory NV, an area of significant unmet need in cancer care.

## Figures and Tables

**Table 1:** Characteristics of the included studies.

Author Year	Country	Cancer Type	Cause of NV	Sample Size	Mean Age (years)	Intervention	Control	Outcome Measures	Main Findings	Adverse Events
Shen 2000 [16]	USA	Breast cancer	Chemotherapy (Cyclophosphamide, cisplatin, carmustine)	104	46	EA + PT	(1) SEA + PT (2) PT	Episodes of vomiting	Adjunct EA was more effective in controlling emesis than minimal needling or antiemetic pharmacotherapy alone although the observed effect had a limited duration.	Minor adverse events (electrical shock sensation, tingling sensation aggravation)
Streitberger 2003 [17]	Germany	Multiple myeloma, breast cancer, hodgkin lymphoma, etc.	Chemotherapy (mainly melphalan), BSCT	80	54	AT + PT	SA + PT	Number of patients with NV, vomiting, episodes of NV	AT has no additional effect for the prevention of acute NV in high-dose chemotherapy.	None observed
Mehling 2007 [18]	USA	Abdominal/pelvic, prostate/testicular, bladder/kidney, breast cancer	Surgery	138	56	AT + Massage	Usual care+ Massage	NRS	A bedside service of massage and AT resulted in decreased pain and depressive mood among postoperative cancer patients when compared with usual care alone.	Not mentioned
Yang 2009 [19]	China	Diverse cancer (not reported)	Chemotherapy	246	51	EA + PT	PT	Effective rate, NRS	EA at ST36 can significantly alleviate the symptoms such as NV caused by chemotherapy of the patients.	Minor adverse events (bleeding around acupoints)
Enblom 2012 [20]	Sweden	Gynecologic, colon, rectal, anal, testicular cancer, etc.	Radiotherapy	324	54	AT + PT	SA + PT	Episodes of NV	AT is not more effective than sham in radiotherapy-induced nausea, but in this study, nearly all patients in both groups experienced that the treatment was effective for nausea.	Minor adverse events (bleeding around acupoints, tiredness, dizziness, needle scratch on skin)
Beith 2012 [21]	Australia	Breast cancer	Chemotherapy (Doxorubicin, cyclophosphamide, others)	32	52	EA + PT	SEA + PT	MAT, episodes of NV, number of patients with NV	EA during chemotherapy is a promising option for controlling the side effects of chemotherapy.	None observed
Zhang 2014 [22]	China	Lung, colon, breast cancer, etc.	Chemotherapy	72	59	EA + PT	SEA + PT	Episodes of NV, complete control rate	EA at PC6 and PC5 combined with Granisetron could obviously relieve nausea in chemotherapy patients at the delay stage.	None observed
McKeon 2015 [23]	Australia	Breast, colorectal, gynecological cancer, etc.	Chemotherapy	60	58	EA + PT	(1) SEA + PT (2) PT	FLIE, NRS, episodes of nausea and vomiting	It was not possible to determine whether EA improved CINV compared to standard care.	Minor adverse events (needling pain, bruising, localized skin irritation)
Rithirangsriroj 2015 [24]	Thailand	Gynecologic cancer	Chemotherapy (Paclitaxel, carboplatin)	70	52	AT + PT	PT	Complete response rate, functional assessment of cancer therapy-general	AT is effective in preventing delayed CINV and promoting a better quality of life with fewer adverse effects.	Minor adverse events (needling pain, soreness)
Wang 2018 [25]	China	Colorectal cancer	Surgery	80	51	EA (1) 1 sessions of treatment (2) 2 sessions of treatment (3) 3 sessions of treatment	No treatment	Number of patients with nausea, vomiting	EA treatment combined with intravenous anesthesia conducted before and after surgery is effective in promoting the recovery of gastrointestinal function in patients undergoing colorectal cancer laparotomy and is obviously better than simple pre-operative EA.	None observed
Wang 2019 [26]	China	Lung cancer	Chemotherapy	140	54	AT + PT (1) before chemotherapy (2) after chemotherapy	PT	Effective rate, Karnofsky performance scale	AT combined with the slow intravenous injection of Tropisetron hydrochloride achieves a satisfactory effect in prevention and treatment. AT before chemotherapy greatly improves the effect on the NV induced by chemotherapy of lung cancer.	Minor adverse events (dizziness)
Li 2020 [27]	China	Breast, ovarian, cervical, endometrial, lung cancer, etc.	Chemotherapy (Cisplatinum, anthracycline, taxane)	134	59	EA + PT	SEA + PT	Complete control rate, common terminology criteria for adverse events	AT as an adjunctive approach could alleviate the severity of CINV compared to the sham control, even though the effect of AT in preventing CINV occurrence is relatively modest.	None observed
Cheng 2020 [28]	China	Lung cancer	Chemotherapy (cisplatin)	100	59	AT + PT (1) before chemotherapy (2) after chemotherapy	PT	Complete control rate, INVR, national cancer institute common toxicity criteria 4.0	AT before chemotherapy can effectively reduce the frequency of NV associated with cisplatin chemotherapy in lung cancer patients, improve the effectiveness of CINV treatment, and be widely promoted in clinics.	Not mentioned
Maeng 2022 [29]	Republic of Korea	Solid tumor	Chemotherapy (Cisplatin, doxorubicin, cyclophosphamide)	42	64	AT + PT	PT	RINVR, MAT	Delayed nausea after highly emetogenic chemotherapy tended to decrease with AT using the RINVR score, though it was also not significant. With the MAT assessment, delayed NV was significantly improved with AT, suggesting a promising effect of AT.	None observed
Bintoro 2022 [30]	Indonesia	Leukemia, renal, liver cancer, etc.	Chemotherapy	60	11	IA + PT	SIA + PT	RINVR	IA treatment is able to reduce the RINVR score and can be applied to pediatric patients because of its minimal side effects.	None observed
Michel-Cherqui 2024 [31]	France	Solid tumor	Chemotherapy	115	64	IA	Usual care	Number of patients with nausea, vomiting, MAT	IA treatment reduces intensity of acute and delayed nausea in patients treated by optimal antiemetic treatment.	Minor adverse events (discomfort)
Shen 2024 [32]	China	Breast cancer	Chemotherapy	239	51	EA + PT	SEA + PT	Complete control rate, VAS	Adding EA to standard triple antiemetic therapy significantly enhances the efficacy of CINV treatment in patients with breast cancer receiving highly emetogenic chemotherapy.	One discontinuation. Minor adverse events (bruising)

Abbreviations—AT, acupuncture treatment; BSCT, blood stem cell transplantation; CINV, chemotherapy-induced nausea and vomiting; EA, electroacupuncture treatment; FLIE, Functional Living Index Emesis tool; IA, intradermal acupuncture; MAT, Multinational Association of Supportive Care in Cancer Antiemesis Tool; NRS, numeric rating scale; NV, nausea and vomiting; PT, pharmacotherapy; RINVR, the Rhodes Index of Nausea, Vomiting, and Retching; SA, sham acupuncture; SEA, sham electroacupuncture, SIA, sham intradermal acupuncture.

**Table 2:** Summary of acupuncture treatment protocols using STRICTA criteria.

Author Year	Names of Acupoints	Number of Needle Insertions	Depth of Insertion (mm)	Needle Type	Needle Stimulation	Needle Retention Time	Number of Treatment Sessions	Treatment Time	Setting and Context of Treatment	Description of Participating Acupuncturists
Shen 2000 [16]	PC6, ST36	4	30~45	36-gauge (Seirin, Shizuoka, Japan)	Electrical (2~10 Hz, <26 mA), De qi (rotation)	20 min	5	2 h before chemotherapy	Inpatient (oncology center at a university medical center)	Acupuncture clinician with 3 and 20 years of acupuncture training
Streitberger 2003 [17]	PC6	2	NM	0.25 × 40 mm (Asia med, Pullach im Isartal, Germany)	Manual, De qi	20 min	2	30 min before chemotherapy	University of Heidelberg	Two trained acupuncturists
Mehling 2007 [18]	PC6, ST36, additional points according to patients	4~14	3~90	34-gauge (Seirin, Shizuoka, Japan)	Manual, De qi	20 min	2	1, 2 days after surgery	University of California, San Francisco	Two licensed acupuncturists
Yang 2009 [19]	ST36	2	25~30	0.30 × 40 mm	Electrical (NM), De qi (rotation)	30 min	10	30 min before chemotherapy	Jilin Hospital at Changchun, China	NM
Enblom 2012 [20]	PC6	2	15	0.30 × 40 mm (Dongbang, Seongnam, Republic of Korea)	Manual, De qi (twirling and lifting)	30 min	12	Before or after the radiotherapy session	Two Swedish university hospitals	Seven acupuncture-trained physiotherapists with experience (range 2–12 years)
Beith 2012 [21]	PC6, ST36, LI4,	4	NM	0.20 × 25 mm (Viva, China)	Electrical (2 Hz, for a 1.5-s duration pulse width), De qi (rotation)	20 min	4	2 h before chemotherapy (each first, second cycles of chemotherapy)	Oncology clinic at the Royal Prince Alfred Hospital, Australia	Two acupuncturists with more than 6 years of training and 10 years of experience
Zhang 2014 [22]	PC6, PC5	4	13~23	0.30 × 25 mm (Jiangsu med, Changzhou, China.)	Electrical (20 Hz, 0~10 mA), De qi	60 min	6	30 min before chemotherapy	Inpatient of first affiliated hospital of China medical university, Zhejiang, China	NM
McKeon 2015 [23]	PC6, ST36, LI4, LR3	8	2~15	0.20 × 30 mm (Sensei, Melbourne, Australia)	Electrical (10 Hz, <10 mA), De qi (thrusting, twirling)	30 min	2	10 min before chemotherapy	Mater Health Services Adults Day Oncology Unit, Brisbane, Australia	A trained acupuncturist with an advanced diploma of acupuncture with 6 years of clinical practice at the time of the study
Rithirangsriroj 2015 [24]	PC6	2	NM	NM	Manual, De qi (rotation)	30 min	2	30 min before chemotherapy	King Chulalongkorn Memorial Hospital Bangkok, Thailand	A third author of this study
Wang 2018 [25]	PC6, ST36, ST37, ST39 (+CV12, ST25)	6	30~70	NM	Electrical (2 Hz, 2~3 mA), De qi (rotation)	30 min	1~3	(1) 1 day before chemotherapy (2) 1 day and 30 min before chemotherapy (3) 1 day, 30 min before, 1 day after chemotherapy	Ningxia Medical University, Yinchuan, China	NM
Wang 2019 [26]	PC6, CV12, ST36	5	20~30	0.25 × 40 mm	Manual, De qi	30 min	3	30 min before or after chemotherapy	Zhejiang Chinese Medical University, China	NM
Li 2020 [27]	PC6, CV12, CV6, ST25, ST36, LR13	10	10–35	0.25 × 40 mm (Ande, Guizhou, China)	Electrical (2/100 Hz), De qi	30 min	6	Twice on the first day of chemotherapy and once consecutively on the following 4 days	Beijing Shijitan Hospital, Beijing Friendship Hospital, Beijing Hospital of Traditional Chinese Medicine, and Guang’ Anmen Hospital	All the acupuncturists who participated in this trial were major in acupuncture for more than 3 years with Chinese medicine practitioner license and had undergone rigorous training in conducting this trial
Cheng 2020 [28]	PC6, ST36	4	NM	0.25 × 40 mm (Andi med, China)	Manual, De qi (twirling, rotating)	30 min	5	30 min before or after chemotherapy	Beijing Traditional Chinese Medicine Hospital	Physicians with 3 years of experience performed the acupuncture for patients
Maeng 2022 [29]	PC6, ST36, LI4, LR3 + (HT8, SP2, LR1, SP1, or GB41, SI5, LI1, ST45)	8	NM	0.25 × 40 mm (Dongbang, Seongnam, Republic of Korea)	Manual, NM	20 min	3	1 h before chemotherapy	Kyung Hee University Hospital	Korean medical doctors
Bintoro 2022 [30]	PC6, ST36	4	NM	NM	Intradermal, NM	3 days	1	Before chemotherapy	Inpatient, Dr. Cipto Mangunkusumo Hospital, Jakarta	NM
Michel-Cherqui 2024 [31]	PC6, diaphragm, additional ear points	5	NM	0.2 × 0.9 mm (semi-permanent needles, Seirin, Shizuoka, Japan)	NM	4 days	1	Before chemotherapy	Multi-center (tertiary-care university hospital/cancer center/general hospital)	Experienced physicians with more than seven years of practice
Shen 2024 [32]	PC6, ST36, LI4	6	NM	NM	Electrical (2 Hz, <10 mA), De qi	45 min	4	Before chemotherapy	Multi-center (six centers in China)	Trained acupuncturists, each with over 3 years of experience in oncology

Abbreviations—AT, acupuncture treatment; BSCT, Blood stem cell transplantation; CINV, chemotherapy-induced nausea and vomiting; EA, electroacupuncture treatment; FLIE, Functional Living Index Emesis tool; IA, intradermal acupuncture; MAT, Multinational Association of Supportive Care in Cancer Antiemesis Tool; NRS, numeric rating scale; NV, nausea and vomiting; PT, pharmacotherapy; RINVR, the Rhodes Index of Nausea, Vomiting, and Retching; SA, sham acupuncture; SEA, sham electroacupuncture, SIA, sham intradermal acupuncture.

## Data Availability

The original contributions presented in the study have been included in the article; further inquiries can be directed to the corresponding authors.

## Appendix A

**Table A1:** The Sites of Acupuncture Points.

PC6	On the anterior aspect of the forearm, between the tendons of the palmaris longus and the flexor carpi radialis, 2 cun proximal to the palmar wrist crease. With the fist clenched, the wrist supinated and the elbow slightly flexed, the two tendons become more prominent. PC6 is located 2 cun proximal to PC7. The posterior point corresponding to PC6 is TE5.
ST36	On the anterior aspect of the leg, on the line connecting ST35 with ST41, 3 cun inferior to ST35. ST36 is located on the tibialis anterior muscle.
CV12	On the upper abdomen, 4 cun superior to the center of the umbilicus, on the anterior median line. CV12 is located at the midpoint of the line connecting the xiphisternal junction and the center of umbilicus.
LI4	On the dorsum of the hand, radial to the midpoint of the second metacarpal bone.
LR3	On the dorsum of the foot, between the first and second metatarsal bones, in the depression distal to the junction of the bases of the two bones, over the dorsalis pedis artery. LR3 can be felt in the depression when moving proximally from LR2 in the gap between the first and second metatarsal bones towards the base of two metatarsal bones.
ST25	On the upper abdomen, 2 cun lateral to the center of the umbilicus.
CV6	On the lower abdomen, 1.5 cun inferior to the center of the umbilicus, on the anterior median line.
LR13	On the lateral abdomen, inferior to the free extremity of the 11th rib. LR13 can be located while the subject is lying on the side with the shoulder flexed. The free extremity of the 11th rib can be palpated below the inferior border of the costal arch.
PC5	On the anterior aspect of the forearm, between the tendons of the palmaris longus and the flexor carpi radialis, 3 cun proximal to the palmar wrist crease. With the fist clenched, the wrist supinated and the elbow slightly flexed, the two tendons become more prominent. PC5 is located 3 cun proximal to PC7.
ST37	On the anterior aspect of the leg, on the line connecting ST35 with ST41, 6 cun inferior to ST35. ST37 is located on the tibialis anterior muscle.
ST39	On the anterior aspect of the leg, on the line connecting ST35 with ST41, 9 cun inferior to ST35. ST39 is located on the tibialis anterior muscle, at the same level as GB35 and GB36.

cun: 1cun is equal to 1 unit which is measured as dividing the height of the human body into 75 equal units.

**Table A2:** Incidence of Adverse Events in Safety Assessment.

		Grade 1 (Mild)		Grade 2 (Moderate)		Grade 3~5	
		Intervention	Control	Intervention	Control	Intervention	Control
Constipation		19	33	0	0	0	0
Diarrhea		2	9	0	0	0	0
Dizziness		8	5	0	0	0	0
Dyspepsia		8	7	0	0	0	0
Electric shock sensation		1	0	0	0	0	0
Headache		9	10	0	0	0	0
Insomnia		2	11	0	0	0	0
Localized bleeding around needle points		78	10	0	0	0	0
Localized skin irritation		5	0	0	0	0	0
Needle scratch		0	10	0	0	0	0
Needling pain		26	0	0	0	0	0
Symptom aggravation							
	Symptoms of CINV (e.g., tingling sensation)	12	1	17	22	0	0
	Increased Alanine transaminase (ALT)	0	1	0	0	0	0
	Increased urea nitrogen and creatinine	1	0	0	0	0	0
Sedation		6	4	0	0	0	0
Tiredness		15	15	0	0	0	0
Not mentioned		0	0	0	0	4	1
Total		192	116	17	22	4	1

Groups: intervention group (11 RCTs, *n* = 620); control group (8 RCTs, *n* = 516).
